# 

**Published:** 1907-05-15

**Authors:** 


CONTENTS

Event and Comment - - - - - - - - - - 217

Mistakes and Failures in Bridge-Work and Some Remedies,
*By Frank E. Logan, D.D.S.* 223

"Pressure" Anesthesia, - - *By Herman Frinz, M.D., D.D.S.* 250

Obituary - - - - - - - - - - - - 255

Indents - - - - - - - - - - - - 256

Announcements - - - - - - - - - - 259

